# Data on the attitudes of nurses and patient׳s family regarding the family presence in the intensive care unit in Birjand, Iran

**DOI:** 10.1016/j.dib.2018.08.202

**Published:** 2018-09-06

**Authors:** Sakineh Ghrbani, Mohammad Hasan Basirinezhad, Fatemeh Heydarinejad, Maryam Salmani mood, Samane Nakhaee, Ali Kavosi

**Affiliations:** a Ghaen Faculty of Nursing and Midwifery, Birjand University of Medical Sciences, Birjand, Iran; b Department of Epidemiology and Biostatistics, School of Public Health, Tehran University of Medical Sciences, Tehran, Iran; c Faculty of Nursing and Midwifery, Birjand University of Medical Sciences, Birjand, Iran; d School of Nursing and Midwifery, Shahroud University of Medical Sciences, Shahroud, Iran; e Medical toxicology and drug abuse research center (MTDRC), Birjand University of Medical Sciences, Birjand, Iran; f Nursing Research Center, Faculty Member, Golestan University of Medical Sciences, Gorgan, Iran

**Keywords:** Attitudes of nurses, Intensive care unit, Birjand, BAVIQ, Patient׳s family

## Abstract

The presence of the patient׳s family on the patient׳s bedside in the intensive care units (ICU) has been a challenging issue among nurses. Therefore, the aim of the data is to evaluate the viewpoints of nurses and the family of patients on the family attendance at the patient bedside in the intensive care units at the educational hospitals in Birjand City. A descriptive cross-sectional study was carried out on 70 nurses working in the intensive care units of the hospitals and 100 members of the family of patients admitted to the intensive care units in 2017. Statistical analysis was carried out by SPSS 16. The findings showed that the average score obtained by nurses and families were 0.46 ± 1.75 and 2.61 ± 0.50, respectively. The data showed that nurses have a negative opinion about the presence of the patients’ family regarding the family presence in the Intensive Care Unit ICU (*P* < 0.001).

**Specifications table**

**Table:** 

Subject area	Nursing and health professions.
More specific subject area	The attitudes of nurses and patient׳s family regarding the family presence in the Intensive Care Unit
Type of data	Tables.
How data was acquired	The demographic information of nurses and the family of patients was collected. The BAVIQ questionnaire and the family-based questionnaire were used to determine the viewpoints of nurses and patients׳ family, respectively.
Data format	Raw, Analyzed.
Experimental factors	The positive and negative beliefs nurses and patient׳s family were analyzed according to the standards to determine the attitudes of nurses and patient׳s family about the family presence in ICU.
Experimental features	The attitudes of nurses and patients׳ family were determined.
Data source location	Educational hospitals of Birjand University of Medical Sciences, Birjand, Iran.
Data accessibility	The data are available with this article
Related research article	Seyyed-Moslem Mahdavi-Shahri, Nurses viewpoint about visiting in coronary care unit. Iranian Journal of Cardiovascular Nursing [3]

**Value of the data**

• Data presented can be used to show that most nurses still have negative views about meeting of patient׳s׳ family in these wards.
• The data demonstrated that nurses believe that visiting patient׳s׳ family reduce the quality of nursing care planning. Therefore, more research is needed to create a suitable work environment for nurses, and as a result, the quality of care planning can be increased.
• The data presented can provide a basis for consideration of the patient׳s needs through communication with the family, which will make it more flexible in the nursing management policy program.

## 1 Data

Table 1, Table 2, Table 3, Table 4, Table 5 summarize the attitudes of nurses and patient׳s family about patient׳s family visit in intensive care units. Nurses with a total score of 1.75 had the same views as the opposition to the meeting. The nurses questioned for the present data believe that the visit is detrimental to the patient׳s privacy and is also a barrier to nurses׳ camaraderie. In addition, nurses believe that physiological stresses and hemodynamic disorders are caused by the visit.

**Table 1:** Frequency distribution of nurses’ responses to negative (N1–N13) and positive directions (N14–N20) in relation to meeting in intensive care unit (ICU).

**Items**	**I completely agree and agree**		**I have no idea**		**I completely disagree and disagree**	
	**Number**	**Percentage**	**Number**	**Percentage**	**Number**	**Percentage**
N1: Family attendance at the patient׳s bedside makes nurses spend more time collecting information for their families and less time for patient care.	70	100	0	0.0	0	0
N2: Disrupts nursing care.	62	88.6	5	701	3	403
N3: An obstacle to nursing care planning.	59	84.3	11	15.7	0	0
N4: prevents the patient fir resting.	41	58.6	21	30.0	8	11.4
N5: Increases the risk of nursing errors.	44	62.9	6	8.6	20	28.6
N6: The presence of the family makes nurses nervous.	47	67.1	17	24.3	6	8.6
N7: Causes unwanted hemodynamic responses in the patient.	42	60	22	31.4	6	8.6
N8: Causes a physiological tension in the patient.	31	44.3	23	32.9	16	22.9
N9: Nurses feel that they are under control.	42	60	15	21.4	13	18.6
N10: Makes psychological pressure in the patient.	24	34.3	20	28.6	26	37.1
N11: An obstacle to jokes between nurses.	41	58.6	12	17.1	17	24.3
N12: This causes family fatigue because they feel they have to be with the patient.	38	54.3	20	28.6	12	17.1
N13: Affects the privacy of the patient.	29	41.4	27	38.6	14	20.0
4N1: Family presence has positive effects on the patient.	44	62.85	20	28.57	6	8.57
N15: Reduces family anxiety.	49	70.0	15	21.42	6	8.57
N16: It is important to improve the patient.	35	50.0	16	22.85	19	27.14
N17: It makes sense for the patient.	37	52.85	22	31.42	11	15.71
N18: It can help in understanding and providing information to the patient.	27	38.57	25	35.71	18	25.71
N19: Improves patient-centered care.	30	42.85	27	38.57	13	18.57
N20: It can have a nursing support aspect.	26	37.14	14	20.0	30	42.85

**Table 2:** The mean, standard deviation for positive and negative attitudes of nurses using one-sample *T* test.

	**Number**	**Mean**	**Standard deviation**	**confidence interval of 95%**		**Significant (*P*) level**
				**Lowest**	**Highest**	
Average of 7 positive phrases	70	2.36	0.67	2.51	2.71	<0.001
Average of 13 negative phrases	70	1.42	0.59	1.28	1.57	<0.001
Average total number of phrases	70	1.75	0.49	1.64	1.86	<0.001

**Table 3:** Frequency distribution of family responses in intensive care units to negative points in relation to meetings in the intensive care units.

**Items**	**I completely agree and agree**		**I have no idea**		**I completely disagree and disagree**	
	**Number**	**Percentage**	**Number**	**Percentage**	**Number**	**Percentage**
F4: My presence increases the likelihood of a hospital infection.	60	60.0	19	19.0	21	21.0
F8: I am afraid I cannot control my feelings during my time at the patient׳s bedside.	69	69.0	3	3.0	28	28.0
F9: Makes nurses spend more time giving me information and less time to care for my patient.	52	52.0	17	17.0	31	31.0
F10: Disrupts nursing care.	50	50.0	9	9.0	41	41.0
F11: The constant presence in the ward makes me tired.	52	52.0	12	12.0	36	36.0
F12: Causes mental stress in my patient.	53	53.0	8	8.0	39	39.0
F13: Disrupts my patient׳s rest.	43	43.0	5	5.0	52	52.0

**Table 4:** Frequency distribution of family responses with patients admitted to the intensive care unit to the items with positive directions regarding appointments in the intensive care units.

**Item**	**I completely agree and agree**		**I have no idea**		**I completely disagree and disagree**	
	**Number**	**Percentage**	**Number**	**Percentage**	**Number**	**Percentage**
F1: I will get more information from my patient and better understand my patient׳s condition.	97	97	0	0.0	3	3
F2: By being present in the ward, I am convinced that the best care is done for my patient.	90	90	2	2	8	8
My presence in the ward helps me better take care of my patient after discharge.	79	79	10	10	11	11
F5: I can provide the nurse with valuable information from my patient.	88	88	7	7	5	5
F6: I will help the nurse to do primary care.	77	77	7	7	16	16
F7: This makes the nurse do a better care of my patient.	83	83	5	5	12	12
F14: My presence in the ward has beneficial and positive effects on the patient.	84	84	3	3	13	13
F15: My anxiety is reduced.	93	93	4	4	3	3
F16: My presence in the ward can be effective in the recovery process.	83	83	6	6	11	11
F17: Improves patient-focused care.	77	77	14	14	9	9
F18: Makes my patient feel more comfortable.	91	91	3	3	6	6

**Table 5:** The mean, standard deviation for positive and negative attitudes of the patients’ in ICU using one-sample *T* test.

	**Number**	**Mean**	**Standard deviation**	**confidence interval of 95%**		**Significant level (*P* > 0.05)**
				Lowest	Highest	
Average of 7 positive phrases	100	1.66	0.91	1.48	1.84	<0.001
Average of 11 negative phrases	100	3.21	0.49	3.12	3.31	<0.001
Average total number of phrases	100	2.61	0.50	2.51	2.71	<0.001

## 2 Experimental design, materials and methods

This data comes from descriptive cross-sectional research that was conducted on all nurses working in ICU and 100 families of patients admitted to the intensive care unit of Valiasr and Imam Reza Hospital in Birjand. The nurses were selected through census sampling method and family of patients admitted to ICU with available sampling method.

### 2.1 Selection criteria for participants in the research

The criteria for entering families to participate included being at least 18 years old, speaking in Persian, having the ability to communicate and being hospitalized for at least 48 h in the above-mentioned wards. The criteria for entering nurses included being employed in intensive care units of the hospitals, having a bachelor׳s degree in nursing and having at least six months of experience in nursing in intensive care unit, willingness to participate in research and completing a questionnaire.

### 2.2 Calculating the sample size

The sample size for the patient׳s family members was determined.

Sample size = $n=\frac{\left(Z_{1-\frac{\alpha }{2}}^{2}\right)S^{2}}{d^{2}}$, as much as 96 subjects that increased to 100 people.

where, *S* = 0.41, *α* = 0.05, and *d* = 0.2.

### 2.3 The data collection tools

1. The Nurses׳ View Point Questionnaire consists of two sections. The first part includes the demographic information registration form for nurses including age, gender, marital status, service record, work experience in the intensive care unit, employment status, and type of shift work. The second part is the BAVIQ questionnaire, which examines the opinions and attitudes of nurses about visits to intensive care units. The validity and reliability of the questionnaire has been confirmed in previous studies [1], [2], [3], [4]. The validity of this questionnaire was evaluated by 10 faculty members of Birjand Nursing and Midwifery Faculty. Its reliability was calculated using Cronbach׳s alpha coefficient up to 0.79. The Likert Score has five options, scoring from 0 to 4. For each of the two positive points, scoring was in the form of "I completely disagree" (0 points), "I disagree" (1 point), "I have no idea" (2 points), "I agree" (3 points), "I completely agree" (4 points). Scoring for each of the points with a negative direction was considered to be the opposite of the positive ones [5], [6]. To obtain the total score of attitudes, the scores of 20 questions of the questionnaire after the alignment of the positive and negative points were summed up and divided by 20. The mean of the numbers obtained was between 0 and 4. The closer the mean to 4 indicates the agreement of the nurses with a free visit. In order to compare the ratio of agreeable and dissenting beliefs, "I agree" and "I completely agree" are considered as agreed groups and "I disagree” and "I completely disagree" are considered as the opposite ones. The item "I do not comment" are excluded from the comparison.
2. The second questionnaire examines the attitude of patients׳ family about the presence of family in ICU, which consists of two parts. The first part contained the demographic data of the patient׳s family included age, gender marital status, educational level, hospitalization ward, hospital location, relativity with the patient, and place of residence. The second part related to the viewpoints of family members of the patients including 18 items. Scoring was calculated based on the five-point Likert scale. For each of the two positive points, scoring was as follows:

"I completely disagree" (0 points), "I disagree" (1 point), "I have no idea" (2 points), "I agree" (3 points), "I completely agree" (4 points). Scoring for each of the points with a negative direction was considered to be the opposite of the positive ones [7], [8]. To obtain the total score of belief, the scores of 18 questions of the questionnaire after the alignment of the positive and negative points were summed up and divided by 18, the mean of the numbers obtained was between 0 and 4. The closer this number to 4 indicates the agreement of the family members of the patients with a free visit. In order to compare the ratio of agreeable and opposite beliefs, "I agree" and "I completely agree" are considered as agreed groups; also, "I disagree" and "I completely disagree" are considered as the opposite ones. Item of "I do not comment" are excluded from the comparison.

After obtaining the research license from the relevant authorities of Birjand University of Medical Sciences and the Ethics Committee, the researcher distributed questionnaires among qualified nurses and family members of patients admitted to intensive care units at various shifts. They were asked to complete the questionnaire without name. The nurses were asked to avoid sharing their personal opinions with other nurses.

Statistical analysis was performed by SPSS Paired Samples *T* Test. The significance level of the tests was considered as significant (*p* < 0.05).
